# Supplementary material for: The GAG-Binding Peptide MIG30 Protects against Liver Ischemia-Reperfusion in Mice
Source: Int J Mol Sci. 2022 Aug 26;23(17):9715. doi: 10.3390/ijms23179715 (PMC9456047; doi:10.3390/ijms23179715)
Supplement: Supplementary file 1 [file ijms-23-09715-s001.zip › ijms-1873321-supplementary.pdf]

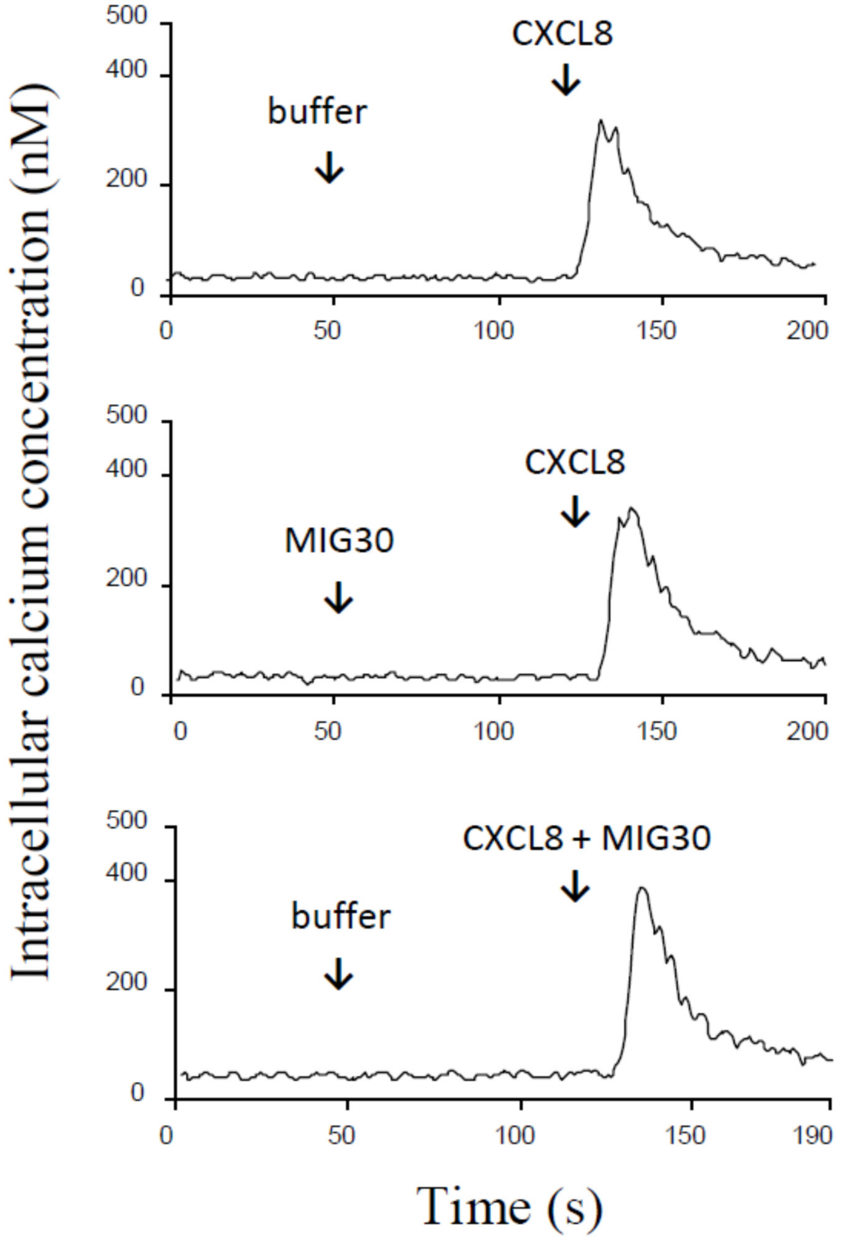

Supplementary Figure S1. MIG30 does not induce nor inhibits CXCL8-induced calcium responses in human neutrophils. The intracellular calcium concentration of purified human neutrophils stimulated with buffer (peptide vehicle) or MIG30 (20 µg/ml), and 70 s later with CXCL8 (10 ng/ml) or the combination CXCL8 and MIG30 (20 µg/ml). The spectra of one representative experiment are shown out of 4 independent experiments. The time of addition of the first and second stimulus are indicated by arrowheads.
